# Late Onset of Estrogen Therapy Impairs Carotid Function of Senescent Females in Association with Altered Prostanoid Balance and Upregulation of the Variant ERα36

**DOI:** 10.3390/cells8101217

**Published:** 2019-10-08

**Authors:** Tiago Januário Costa, Francesc Jiménez-Altayó, Cinthya Echem, Eliana Hiromi Akamine, Rita Tostes, Elisabet Vila, Ana Paula Dantas, Maria Helena Catelli de Carvalho

**Affiliations:** 1 Department of Pharmacology, Institute of Biomedical Sciences, University of Sao Paulo, Sao Paulo 05508-900, Brazil; tiago.januario@uol.com.br (T.J.C.); cinthya.echem@gmail.com (C.E.); eliakamine@usp.br (E.H.A.); mhccarvalho@uol.com.br (M.H.C.d.C.); 2 Facultat de Medicina, Departament de Farmacologia, Terapèutica i Toxicologia, Institut de Neurociències, Universitat Autònoma de Barcelona, 08193 Bellaterra, Spain; Francesc.Jimenez@uab.cat (F.J.-A.); elisabet.vila@uab.cat (E.V.); 3 Group of Atherosclerosis and Coronary Disease, Institut Clinic del Torax, Institut d’Investigaciones Biomédiques August Pi I Sunyer (IDIBAPS), 08036 Barcelona, Spain; 4 Pharmacology Department, Ribeirao Preto Medical School, University of Sao Paulo, Sao Paulo 14049-900, Brazil; rtostes@usp.br

**Keywords:** estrogen, aging, estrogen receptor variants, cerebrovascular function, menopause, hormone replacement therapy

## Abstract

Recent analysis of clinical trials on estrogen therapy proposes the existence of a therapeutic window of opportunity for the cardiovascular benefits of estrogens, which depend on women’s age and the onset of therapy initiation. In this study, we aimed to determine how vascular senescence and the onset of estrogen treatment influence the common carotid artery (CCA) function in senescent and non-senescent females. Ovariectomized female senescence-accelerated (SAMP8) or non-senescent (SAMR1) mice were treated with vehicle (OVX) or 17β-estradiol starting at the day of ovariectomy (early-onset, E_2_E) or 45 days after surgery (late-onset, E_2_L). In SAMR1, both treatments, E_2_E and E_2_L, reduced constriction to phenylephrine (Phe) in CCA [(AUC) OVX: 193.8 ± 15.5; E_2_E: 128.1 ± 11.6; E_2_L: 130.2 ± 15.8, *p* = 0.004] in association with positive regulation of NO/O2- ratio and increased prostacyclin production. In contrast, E_2_E treatment did not modify vasoconstrictor responses to Phe in OVX-SAMP8 and, yet, E_2_L increased Phe vasoconstriction [(AUC) OVX: 165.3 ± 10; E_2_E: 183.3 ± 11.1; E_2_L: 256.3 ± 30.4, *p* = 0.005]. Increased vasoconstriction in E_2_L-SAMP8 was associated with augmented thromboxane A2 and reduced NO production. Analysis of wild-type receptor alpha (ERα66) expression and its variants revealed an increased expression of ERα36 in E_2_L-SAMP8 in correlation with unfavorable effects of estrogen in those animals. In conclusion, estrogen exerts beneficial effects in non-senescent CCA, regardless of the initiation of the therapy. In senescent CCA, however, estrogen loses its beneficial action even when administered shortly after ovariectomy and may become detrimental when given late after ovariectomy. Aging and onset of estrogen treatment are two critical factors in the mechanism of action of this hormone in CCA.

## 1. Introduction

The female aging process and associated disorders may be linked to changes in the hormonal milieu [1]. Of all aging-related disorders, cardiovascular disease is the leading cause of morbidity and mortality in older women [2]. Although estrogen modulates several pathways that are closely associated with aging, it remains unknown whether estrogen can delay cardiovascular senescence, or how estrogen-mediated responses occur in a senile vasculature.

In 1985, the observational study Nurses’ Health Study reported that estrogen therapy reduces the risk of cardiovascular disease in postmenopausal women [3]. In agreement with these clinical data, many experimental studies demonstrated that estrogen has beneficial effects in several signaling pathways in the vascular system [4,5,6,7,8,9]. However, double-blind, randomized clinical trials—such as Woman’s Health Initiative (WHI) and Heart and Estrogen/progestin Replacement Study (HERS I and II)—showed that estrogen therapy increases cardiovascular risk, and more specifically the risk for stroke in postmenopausal women [10,11]. Stroke is the leading cause of prolonged disability and the third leading cause of death among women [2]. Despite this, there is still a concerning gap in the comprehension of the mechanisms involved in the estrogen regulation of the cerebrovascular function of aged women.

Among the explanations for the discrepancies between clinical trial and observational studies, is the fact that women included in these trials were on average 60 years of age when they started receiving estrogen therapy, typically more than 10 years after the onset of menopause. While in the first observational study, women were younger, approximately 50 years of age, and closer to menopause when they initiated hormonal therapy. Detailed analysis by aging groups suggests that early initiation of estrogen therapy produces more favorable results than the average of late-onset used in most trials [12]. These analyzes led scientists to conceive the so-called “timing hypothesis”, which relies on the concept that estrogen has beneficial effects if taken before, or close to, the onset of menopause when the detrimental aging effects or lack of estrogen in the vasculature have not yet been established [13]. In 2016, the clinical trial ELITE (Early versus Late Intervention Trial with Estradiol) strengthened this theory by showing that estrogen therapy is associated with less progression of subclinical atherosclerosis only when it is initiated within six years after menopause onset [14]. Aging per se is known to cause a series of alterations in the endogenous mechanisms that control vascular function, leading to subsequent increased risk of cerebrovascular disease [15]. Moreover, vascular aging can alter estrogen-mediated signaling pathways [16] and, as a consequence, estrogen therapy may become harmful rather than beneficial to the vasculature.

The classical effects of estrogen in the vasculature are mediated via interactions with two subtypes of nuclear receptors belonging to a superfamily of hormone-inducible transcription factors (ERα and ERβ), and by a seven-transmembrane G protein-coupled ER, termed GPER, associated to activate rapid signaling cascades after estrogen binding (for review, see [17]). Although the three estrogen receptors are known to promote cardiovascular effects, ERα is the most associated with the beneficial effects of estrogen in the endothelial and smooth muscle cells [17]. Furthermore, molecular studies have identified and cloned alternative splicing variants of ERα, which have been associated with modifications in the activation of the major signaling pathways by this receptor [17,18,19]. These splicing variants of ERα dimerize with the wild-type 66Kd receptor (ERα66), and may serve as a competitive inhibitor of ERα66 binding to DNA [18,19] and, therefore, an increase of ERα splicing may interfere with ERα66 transcriptional activity function. Upregulation of this variant has been associated with rapid estrogen and anti-estrogen signaling in gynecological cancer [20], although little is known about their correlation with cardiovascular disease.

In this study, we interrogate the complicated relationship between the timing of estrogen therapy and senescence in the protection or detriment of female cerebrovasculature. The common carotid arteries (CCA) carry the main supply of blood to the brain, and inadequate circulation of blood through these vessels may result in cerebrovascular events. We hypothesized that vascular senescence and the onset of estrogen treatment modifies arterial function in association with changes in the expression of variants of classical ERα. For this purpose, we analyzed vascular reactivity of the CCA from ovariectomized (OVX) senescence-accelerated prone (SAMP) mice following early- or late-onset of estrogen treatment. Our previous studies have established OVX-SAMP as a model that closely mimics menopause in women, as it can concomitantly determine vascular changes by estrogen withdrawal (or replacement) in a senescent environment [21].

## 2. Material and Methods

### 2.1. Animal Models and Hormonal Treatment

Female senescence-prone inbred (SAMP8, aging model n = 80) and senescence-resistant inbred strain (SAMR1, young control *n* = 80) mice, were obtained from the breeding stock at Parc Cientific de Barcelona. The senescence-accelerated mouse model was developed as a result of selective inbreeding of mice showing a phenotype of severe exhaustion (SAM-prone) and inbreeding of a normal phenotype (SAM-resistant). SAMP strains are known to manifest spontaneously various pathobiological phenotypes, including vascular senescence. SAM offers several advantages in aging cardiovascular research as it ages fast and predictably, allowing the execution of experimental work in a convenient and standard time course [21]. The animals were housed at the Animal Facility of the University of Barcelona according to institutional guidelines (constant room temperature 22 °C, 12-h light/dark cycles, 60% humidity, standard mice chow, and water ad libitum). All the procedures used in this study were approved and performed following the guidelines of the Ethics Committee of the University of Barcelona (Protocol 272/12), the Institute of Biomedical Sciences, University of São Paulo (ICB-USP—Protocol 64, page 20, book 3. 27.05.2014), and in agreement with the *Guide for the Care and Use of Laboratory Animals* published by the US National Institute of Health (NIH Publication No.85-23, revised 1996). At six months of age, SAMR1 and SAMP8 mice were ovariectomized under controlled inhalant anesthesia with isoflurane (4% induction and 1.5–2% maintenance). After ovariectomy, mice were divided into three groups: (1) ovariectomized treated with vehicle (OVX); (2) early onset of estrogen treatment, initiated in the first day of ovariectomy (E_2_E); and (3) late onset of estrogen treatment, initiated 45 days after ovariectomy (E_2_L). Cyclic estrogen therapy (5 µg/kg of 17β-estradiol diluted in mineral oil), was administrated by subcutaneous injections every third day in order to provide a more physiological hormonal milieu [16]. The efficacy of ovariectomy and estrogen treatment was determined by the uterine weight and plasma estrogen concentrations. Sixty days following the ovariectomy, all mice were euthanized with sodium pentobarbitone (85 mg/Kg, I.P.) and the CCAs were dissected and kept in ice-cold physiological salt solution and prepared for different experiments mainly as described [22].

### 2.2. Vascular Function Study

Segments (2 mm) of CCA with intact endothelium were mounted on an isometric wire myograph (model 410 A; J.P. Trading, Aarhus, Denmark), as previously described [22]. The myograph was filled with modified Krebs solution ((in mM): NaCl 130; NaHCO_3_ 14.9, KCl 4.7, KH_2_PO_4_ 1.18, MgSO_4_ 1.17; CaCl_2_.2H_2_O 1.56, EDTA 0,026 and glucose 5.5), and kept at 37 °C, 95% O_2_ and 5% CO_2_. After 60 min (min) of equilibration, CCA segments were stimulated three times (10-min interval) with a KCl 60 mM solution until the contraction reached a stable plateau (~15 min). After washout and return to a stable baseline, consecutive concentration–response curves—with acetylcholine (ACh, 10^−10^ to 3 × 10^−5^ M), phenylephrine (Phe, 10^−9^ to 10^−5^ M), and sodium nitroprusside (SNP, 10^−10^ to 3 × 10^−5^ M)—separate by washout and 30 min intervals, were performed in the absence or the presence of several inhibitors. In some experiments, contraction to the thromboxane A_2_ analog (U46619, 10^−9^ to 10^−5^ M), instead of Phe was performed. Vasodilation to ACh and SNP were performed in U46619 (10^−7^ M) pre-contracted vessels. The contribution of the different endothelium-derived factors to the vascular responses was determined by treating isolated CCA segments with one of the following inhibitors: (1) nonselective nitric oxide synthase (NOS) inhibitor Nω-nitro-L-arginine methyl ester (L-NAME; 10^−4^ M); (2) O_2_^−^ scavenger (Tempol, 10^−5^ M); (3) non-selective COX inhibitor (Indomethacin, 10^−6^ M); (4) selective COX-1 inhibitor (SC560, 10^−5^ M); or (5) selective COX-2 inhibitor (NS398, 10^−6^ M). Experiments were done in parallel with segments from the same animal. Contractions to Phe and U46619 are shown as a percentage (%) of the contractile response to 60mM KCl. Relaxations to ACh and SNP were expressed as the percentage (%) of U46619 precontraction. The area under the concentration–response curve (AUC) was used as an overall measure of cumulative responses induced by agonists in the presence of vehicle or specific inhibitors.

### 2.3. Quantitative Real-Time PCR (qPCR) for Detection of COX and eNOS Expression

Total RNA was isolated from CCA using TRizol^®^ reagent (Sigma-Aldrich, St Louis, MI, USA) according to the manufacturer’s instructions. The amount of mRNAs encoding the COX isoforms (COX-1 and COX-2) and downstream enzymes (PGI2S and TXA2S), as well as eNOS, were quantified by qPCR based on SYBR^®^ Green fluorescence (Applied Biosystems, Carlsbad, CA, USA). β-actin was used as an internal control. Primer sequences are described in Table S3 (Major Resources Tables). qPCR reactions were performed, recorded, and analyzed using the Corbett Research system (Corbett Life Sciences, Sydney, Australia). The conditions for qPCR were as follows: 95 °C for 2 min, 40 cycles of 95 °C for 15 s (s), and 60 °C for 1 min. Cycle threshold (Ct) values obtained for each gene were referenced to β-actin (ΔCt) and converted to the linear form using the term 2^−ΔΔCt^ as a value directly proportional to the copy number of complementary DNA and the initial amount of mRNA.

### 2.4. Measurement of Prostanoids Release from Vascular Segments

The release of prostanoids following Phe stimulation was determined by enzyme immunoassay (ELISA). Aliquots of Krebs–Henseleit solution were collected after the concentration–response curves to Phe and kept at room temperature for 30 min for complete hydrolysis of prostacyclin (PGI_2_) and thromboxane A_2_ (TXA_2_) into their stable metabolites, 6-Keto-PGF_1α_ and TXB_2_, respectively. Analysis of specific contributions of COX-1 and COX-2 to prostanoids release was carried out in organ baths containing vehicle-treated CCA or CCA treated with selective inhibitors of COX-1 or COX-2. Levels of 6-Keto-PGF_1α_ and TXB_2_ were assessed in duplicate and calculated according to the ELISA kit manufacturer (Cayman Chemical, Ann Arbor, MI). The results were expressed as pM for vehicle-treated CCA and as Log2 of fold change in arteries treated with selective inhibitors of COX-1 (SC396) or COX-2 (NS398).

### 2.5. Western Blot Analysis

SDS-PAGE resolved equal amounts of protein from each CCA sample (30 μg) on 4–12% gels and electroblotted onto nitrocellulose membranes. Membranes were incubated for 1 h (h) with casein-based blocking buffer, following overnight incubation at 4 °C with specific primary antibodies as follows: polyclonal rabbit anti-ERα36, 1:1000 (Alpha Diagnostic, San Antonio, TX, USA); polyclonal rabbit anti-ERα66 1:1000 (Santa Cruz Biotechnology, Sta Cruz, CA, USA). After washes, membranes were incubated with specific horseradish peroxidase-labeled secondary antibodies in phosphate-buffered saline (PBS) containing 1% casein-based buffer. Following additional washes, the chemiluminescent signal was visualized by LAS4000 imaging system (GE Healthcare, Chicago, IL, USA). All membranes were reblotted using a mouse monoclonal antibody anti-α-actin (1:2000; Agilent Dako, Sta Clara, CA, USA) as the loading control. Densitometric analyses of western blots were performed using ImageJ software, and data were normalized to corresponding values of αActin densitometry.

### 2.6. Methylation Status of Gene Encoding ERα and Expression of Splicing Variants

The methylation status of different CpG enriched regions of the gene encoding ERα was determined by methylation-sensitive qPCR, as described [16]. Following mock digestion or enzymatic digestion with HpaII (methylation-sensitive) or MspI (no methylation-sensitive), resulting DNA was amplified using qPCR with Power SYBR^®^ Green master mix, as described by the manufacturer (Applied Biosystems). PCR primers were designed using Methyl Primer Software (Applied Biosystems) to amplify five distinct CpG enriched regions of ERα gene or to amplify a region that is devoid of any of the restriction sites of the enzymes used in the design of the experiment, as an internal control (Table S4, Major Resources Tables). Methylation at CpG sites prevents HpaII, but not MspI, digestion and allows the amplification of the fragment, resulting in a low cycle threshold (Ct) value. In contrast, if the CpG island is not methylated, HpaII cleaves DNA and prevents the amplification of the fragment, resulting in higher Ct values. The percentage (%) of methylation of a given site was determined from the change in Ct value (ΔCt) of methylated-sensitive and mock digested samples. Taking into account the fundamental principle that each successive round of PCR amplification results in approximately a 2-fold increase in the amount of product, the % Methylation was calculated as 100 × (2^−0.7(ΔCt)^). Each sample was analyzed in duplicate. The expression of wild type ERα and C-terminally truncated ERα product (CTERP) was determined in mice CCA by SYBR green-based qPCR. For relative quantification, the amount of CTERP splicing expressed in mice CCA was normalized by the expression of the very same wild type gene (ERα) and the very same sample, as previously described.

### 2.7. Data Analysis and Statistics

Data are expressed as mean ± SEM of the number (n) of mice indicated in the figure legends. The extra sum-of-squares F determined the differences in the fit of concentration–response curves in all groups. The AUC was calculated for individual contractile or relaxing concentration–response curves and expressed as arbitrary units. The contribution of different endothelium-derived factors to Phe-induced contractions was calculated by subtracting the AUC for Phe curves in the presence of inhibitors from the AUC for control Phe curves (ΔChange). Brown–Forsythe testing determined equality in variances among experimental groups (OVX, E_2_E, and E_2_L). One-way ANOVA analyzed the dependence of data on the onset of estrogen therapy (none, early, or late) in SAMR1 or SAMP8 with Bonferroni’s post-test. The analysis was carried out using the Prism software (GraphPad Software V7.0, San Diego, CA, USA), and statistical significance was accepted at *p* < 0.05.

## 3. Results 

### 3.1. Basic Parameters for Ovariectomy and Estrogen Treatment Efficacy

Ovariectomy resulted in reduced uterine weight and plasma 17β-estradiol concentrations in SAMR1 and SAMP8 female mice (data not shown). Those parameters were restored by both early (E_2_E) and late (E_2_L) onset of 17β-estradiol treatment (Table 1).

### 3.2. Early- and Late-Onset Estrogen Treatments Promote Different Vasoconstrictor Effects in Response to Adrenergic Stimulus in SAMR1 and SAMP8 Ovariectomized Female Mice

**Figure 1:**
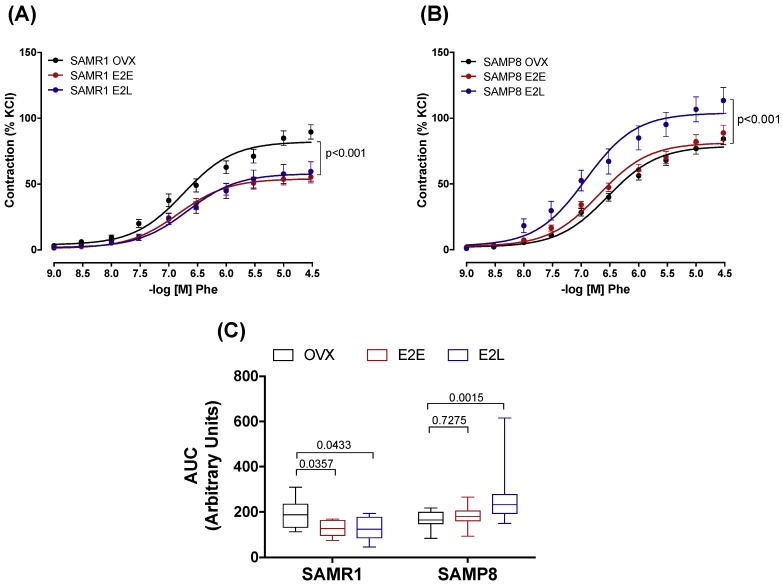
Phenylephrine-induced contractions. Concentration–effect curves to Phenylephrine (Phe) determined in endothelium-intact common carotids arteries from SAMR1 (**A**) and SAMP8 (**B**) female mice. Curves were obtained in vessels from untreated ovariectomized mice (OVX), and ovariectomized mice receiving early-onset (E_2_E) or late-onset (E_2_L) of 17β-estradiol treatment. Differences in vascular responses are expressed as the area under the curve (AUC) (**C**). Each point of the curve and bar graphs represents the mean ± SEM from 10–18 independent experiments. The extra sum-of-squares F. One-way ANOVA determined differences in the fit of concentration–response curves analyzed the dependence of data on the onset of estrogen therapy (none, early, or late) in SAMR1 or SAMP8 with Bonferroni’s post-test. *p*-values and comparisons are expressed next to the curves and on top of bar graphs. Significance is considered when *p* < 0.05.

To determine how the onset of estrogen therapy affects the vascular function of senescent and non-senescent females, we tested the responses of CCA to different vasoactive agents. All agonists studied induced concentration-dependent responses in CCA rings in either group. In SAMR1, treatments with either E_2_E or E_2_L reduced the contractile response to Phe, compared to untreated OVX (Figure 1A), as demonstrated by the smaller area under the curves (Figure 1C). In SAMP8, responses to Phe after E_2_E treatment were similar to those observed in the OVX group. However, E_2_L treatment increased Phe vasoconstriction when compared to the OVX group (Figure 1B,C). On the other hand, there were no differences in the vasoconstrictor responses to the thromboxane A_2_ analog U46619 (Figure S1). Neither did we observe significant differences in vasodilatation responses to both endothelium-dependent (ACh) and endothelium-independent (SNP) agents (Figure S2).

### 3.3. Estrogen Differently Regulates the Contribution of NO/Superoxide Anion (O_2_^−^) Pathways to Phe Contraction in Non-Senescent (SAMR1) and Senescent (SAMP8) Carotids

**Figure 2:**
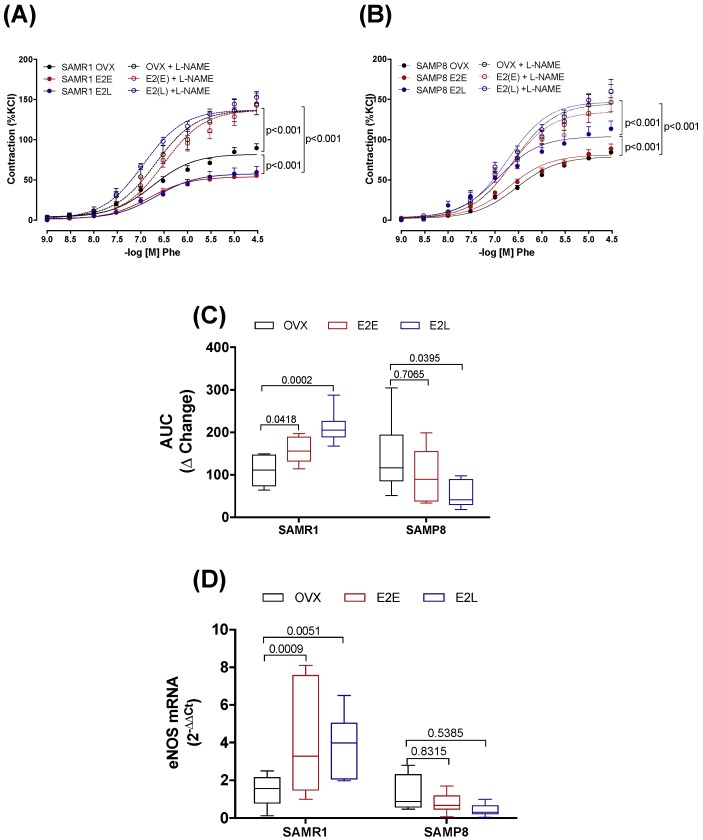
Role of NO in phenylephrine-induced contractions. Concentration–response curves to phenylephrine (Phe) in endothelium-intact common carotid artery from SAMR1 (**A**) and SAMP8 (**B**). Arteries were isolated from untreated ovariectomized mice (OVX), or OVX treated with early-onset of 17β-estradiol treatment (E_2_E) or late-onset 17β-estradiol treatment (E_2_L). Curves were obtained in the absence or the presence of L-NAME (10^−4^ M). The contribution of NO to Phe contraction is expressed as the change (Δ Change) in the area under the curve in L-NAME treated arteries relative to untreated arteries (**C**). mRNA levels of eNOS (**D**) normalized by β-actin mRNA and relative to the expression of untreated OVX mice. Each point represents the mean ± SEM from 8–17 independent experiments. The extra sum-of-squares F determined differences in the fit of concentration–response curves. One-way ANOVA analyzed the dependence of data on the onset of estrogen therapy (none, early or late) in SAMR1 or SAMP8 with Bonferroni’s post-test. *p*-values and comparisons are expressed next to the curves and on top of bar graphs. Significance is considered when *p* < 0.05.

Inhibition of NO production by L-NAME increased the vasoconstrictor responses to Phe in all groups of SAMR1 (Figure 2A) and SAMP8 (Figure 2B) female mice. The ΔChange in the AUC (Figure 2C) shows that there is a more significant contribution of NO to attenuate contractile responses in arteries of non-senescent females (SAMR1) treated with both E_2_E and E_2_L than in untreated OVX-SAMR1. In SAMP8, the ΔChange in the AUC of E_2_E was similar to untreated mice. In opposition, E_2_L displayed a lower ΔChange in the AUC compared to untreated OVX-SAMP8, suggesting a smaller contribution of NO to Phe contraction in this group (Figure 2C). Variations in the contribution of NO to vascular reactivity could be related to changes in both NO production, either via eNOS upregulation or downregulation, and modulation of NO scavenging by O_2_^−^. Analysis of mRNA expression showed an increase in eNOS expression in estrogen-treated groups in SAMR1, but not in SAMP8 (Figure 2D). The influence of O_2_^−^ on Phe-induced contractions was assessed by performing contractile curves in the presence of O_2_^−^ scavenger, tempol. Incubation with tempol decreased Phe contractions in CCA of non-senescent untreated OVX, without affecting vasoconstrictor responses in estrogen-treated SAMR1 (Figure S3). In senescent females, scavenging of O_2_^−^ by tempol decreased contractions to Phe in all SAMP8 groups (Figure S3).

### 3.4. Senescence and Onset of Estrogen Therapy Play an Important Role in Regulating the Contribution of Vasodilator and Vasoconstrictor Prostanoids to Phe Contraction

**Figure 3:**
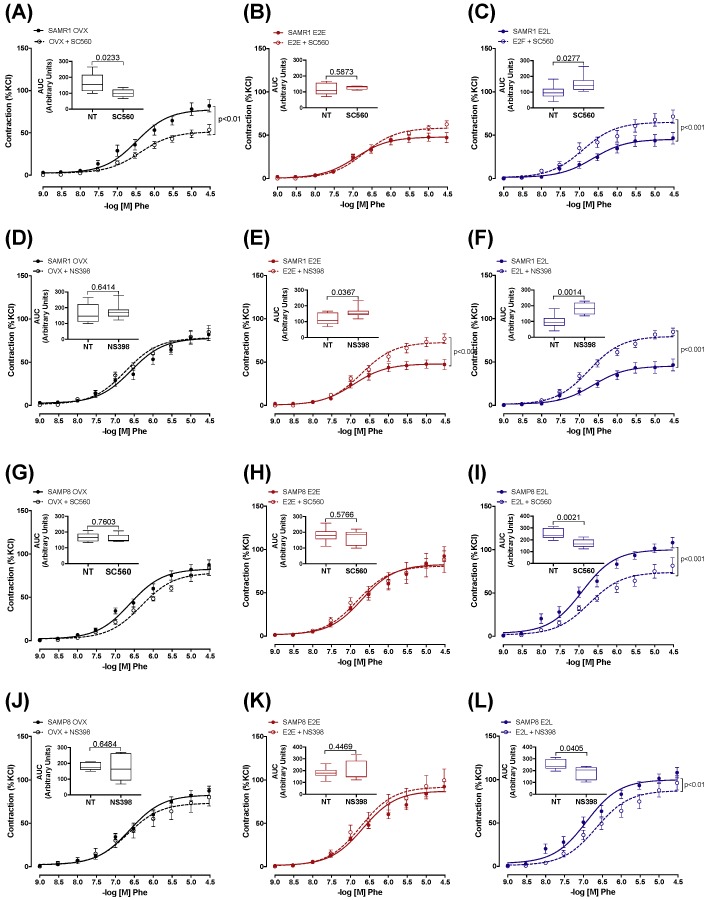
Role of COX-derived prostaglandins in phenylephrine-induced contractions. Concentration–response curves to phenylephrine (Phe) in the endothelium-intact common carotid artery from SAMR1 (**A**–**F**) and SAMP8 (**G**–**L**). Arteries were isolated from untreated ovariectomized mice (OVX) or treated with early-onset of 17β-estradiol treatment (E_2_E) or late-onset 17β-estradiol treatment (E_2_L), as indicated. Curves were obtained in the absence or the presence of COX-1 (SC560, 10^−6^ M) or COX-2 (NS 398, 10^−6^ M) inhibitor. Inset graphs represent the areas under the curve (AUC) obtained from the curves of untreated carotids (NT) or those receiving selective COX inhibition. Each point represents the mean ± SEM from 5–10 independent experiments. The extra sum-of-squares F determined differences in the fit of concentration–response curves. One-way ANOVA analyzed the dependence of data on the onset of estrogen therapy (none, early, or late) in SAMR1 or SAMP8 with Bonferroni’s post-test. *p*-values and comparisons are expressed next to the curves and on top of bar graphs. Significance is considered when *p* < 0.05.

**Figure 4:**
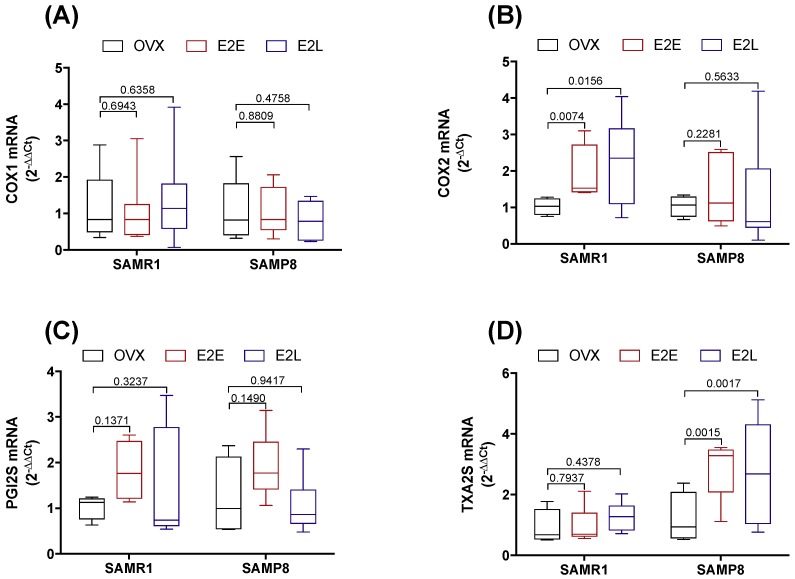
mRNA expression in cyclooxygenase cascade. COX-1 (**A**), COX-2 (**B**), PGI2S (**C**), and TXA2S (**D**) expression in common carotid artery of SAMR1 and SAMP8 normalized by β-actin mRNA and relative to the expression of untreated OVX mice. Total RNA was obtained in vessels from untreated ovariectomized female mice (OVX), OVX mice receiving early-onset 17β-estradiol treatment (E_2_E), and OVX mice receiving late-onset 17β-estradiol treatment (E_2_L). Each point represents the mean ± SEM from 5–10 independent experiments. One-way ANOVA analyzed the dependence of data on the onset of estrogen therapy (none, early, or late) in SAMR1 or SAMP8 with Bonferroni’s post-test. *p*-values and comparisons are expressed on top of bar graphs. Significance is considered when *p* < 0.05.

We next sought to determine the contribution of prostanoids metabolites to the regulation of Phe contraction by estrogen, and how their contributions are affected by vascular senescence and therapy onset. Accordingly, CCAs were treated with the unspecific inhibitor of cyclooxygenases (COX) indomethacin. Pre-incubation with indomethacin decreased Phe contraction in estrogen-untreated OVX-SAMR1 and all three groups of SAMP8 (Figure S4), suggesting a predominance of COX-derived vasoconstrictor metabolites. The specific role of COX isoenzymes to Phe contractions was then determined by treating CCA with the selective inhibitors of COX-1 (SC560) or COX-2 (NS398). In SAMR1, COX-1, but not COX-2, inhibition reduced vasoconstrictor responses in untreated OVX, indicating a contribution of COX-1-derived vasoconstrictor prostanoids to Phe-induced contractions (Figure 3A,D). On the contrary, Phe contraction in E_2_E was modulated by a COX-2-derived vasodilator (Figure 3B,E). Late-onset of estrogen treatment (E_2_L) modified prostanoids’ contribution to Phe vasoconstriction by increasing both COX-1- and COX-2-derived vasodilators (Figure 3C,F). In the senescent CCA from SAMP8, both COX inhibitors reduced vasoconstriction to Phe only in the E_2_L group (Figure 3I,L). Inhibition of either COX-1 or COX-2 did not affect contractions in arteries form untreated OVX-SAMP8 (Figure 3G,J) or SAMP8 E_2_E (Figure 3H,K). mRNA expression analysis of the major enzymes involved in the prostanoids’ biosynthesis cascade revealed differential regulation by estrogen in non-senescent and senescent CCA (Figure 4). Estrogen therapy increased the expression of COX-2 in SAMR1 (Figure 4B) and augmented the expression of TXA_2_ synthase in SAMP8 (Figure 4D). In contrast, no changes in the expression of COX-1 (Figure 4A) or PGI_2_ synthase (Figure 4C) were observed.

### 3.5. Dual Effect of COX-1 and COX-2 in Prostanoids Production

Prostanoids release from CCA was determined in the Krebs–Henseleit solution collected after concentration–response curves to Phe. In SAMR1, E_2_E increased the release of PGI_2_ by ~2-fold, while it decreased the TXA_2_ released by Phe by ~3-fold in comparison to untreated OVX. Late-onset estrogen therapy did not produce the inhibitory effects of estrogen on TXA_2_ release in untreated OVX-SAMR1, even though it increased PGI_2_ concentration. In senescent CCA, neither early nor late-onset estrogen treatments modified PGI_2_ release (Supplementary Figure S5), while E_2_L increased TXA_2_ concentration induced by Phe stimulation (Supplementary Figure S6). Selective inhibition of COX-1 and COX-2 produced a differential regulation of prostanoids biosynthesis depending on the onset of estrogen therapy in non-senescent and senescent arteries. In SAMR1, increased release of PGI_2_ by E_2_E and E_2_L was mainly dependent on COX-2, since the degree of change in PGI_2_ production (Log Fold Change) upon estrogen treatment was only observed when the CCAs were inhibited with NS398. In SAMP8, both COX-1 and COX-2 inhibitors similarly decreased PGI_2_ production in all groups (Figure S5). As for TXA_2_, COX-1 is the primary regulator of its production in CCA of OVX-SAMR1 (Figure S6). On the other hand, the increase in TXA_2_ observed upon E_2_L treatment in SAMP8 was regulated by COX-2 (Figure S6).

### 3.6. Late-Onset Estrogen Treatment Increases Alternative Splicing of Estrogen Receptor Alpha (ERα36) in Aging

**Figure 5:**
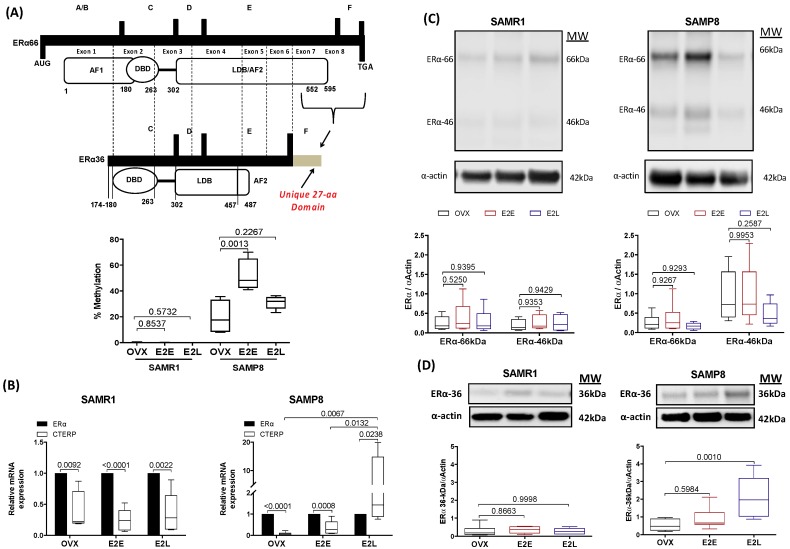
Transcriptional and post-transcriptional regulation of ERα. Percentage of specific DNA methylation in the gene encoding ERα in common carotid artery of SAMR1 and SAMP8 (**A**). Bar graphs show the percentage (%) of methylation in the region comprised between exon 7 and exon 8 and translated to the C-terminus. mRNA expression of C-terminally truncated ERα product (CTERP) relative to the expression of wild-type (ERα66) in mice carotid (**B**). Protein expression of the classical ERα66 and the alternative splicing variants ERα46 (**C**) and ERα36 (**D**) in common carotid normalized by the expression of α-actin. Samples of genomic DNA, total RNA or protein were obtained in vessels from untreated ovariectomized mice (OVX), ovariectomized mice under early-onset (E_2_E) or late-onset (E_2_L) of 17β-estradiol treatment. Each point represents the mean ± SEM from 5–7 independent experiments. One-way ANOVA analyzed the dependence of data on the onset of estrogen therapy (none, early, or late) in SAMR1 or SAMP8 with Bonferroni’s post-test. *p*-values and comparisons are expressed on top of bar graphs. Significance is considered when *p* < 0.05.

Most of the vascular protective effects of estrogens are mediated through interaction with ERα. We next evaluated how senescence and therapy onset modify ERα expression. Analysis of methylation sites in the gene encoding ERα by methylation-sensitive quantitative PCR showed a marked increase of methylation in the region corresponding to Exon 8 in senescent arteries (Figure 5A). The early onset of estrogen treatment further increased the degree of methylation in the CAA of OVX-SAMP8. In order to determine whether changes in the degree of methylation of the ERα gene can affect the pattern of receptor expression, we analyzed mRNA and protein expression of wild-type ERα and its alternative splicing. The onset of estrogen therapy did not change gene (Supplementary Figure S7) or protein (Figure 5C) expression of the wild-type ERα (ERα-66kDa) in CCA. Gene expression of the variant splicing ERα-36kDa was marked lower relatively to the wild-type receptor in most groups, except SAMP8 E_2_L, which exhibited a 6-fold increase over the expression of the wild type ERα (Figure 5B). Changes in mRNA expression of ERα-36kDa corresponded to the pattern of protein expression (Figure 5D). No changes in the expression of the variant ERα-46kDa were found (Figure 5C).

## 4. Discussion

In this study, we describe that the onset of estrogen therapy modifies the vascular function of ovariectomized senescent female mice (SAMP8). Our findings are consistent with new clinical studies suggesting that the beneficial effects of estrogen therapy in the cardiovascular system depend on the time when therapy is initiated [14]. Our data show that estrogen reduces constriction to the adrenergic stimulus by Phe in the CCA of non-senescent OVX mice, regardless of treatment onset, by favoring the release of endothelium-derived relaxing factors, such as NO and PGI_2_. Nonetheless, onset timing plays a critical role when arteries are senescent, a more representative model of arteries in menopausal women. In CCA of senescent female mice (SAMP8), late-onset of estrogen treatment increases adrenergic vasoconstriction in association with augmented TXA_2_ production and upregulation of ERα36 expression, alternative splicing of the classical ERα.

Estradiol, also known as E_2_ or 17β-estradiol, has been widely used as a therapeutic choice for postmenopausal women seeking amelioration of many different menopause-associated symptoms, including vasomotor symptoms, vaginal atrophy, and prevention of osteoporosis [23]. Because premenopausal women are less likely to progress to cardiovascular disease, a protective cardiovascular effect by estrogen was proposed in the past involving a myriad of different mechanisms [24]. Nevertheless, important randomized and placebo-controlled trials—including the WHI and HERS—raised doubts and concerns about the use of estrogen in the primary and secondary prevention of cardiovascular disease [10,11]. More importantly, the WHI study alerted about the increased risk of stroke not offset by the lower risk of coronary heart disease. The surprising controversies on the risks and benefits of estrogen therapy are, in part, associated with the time of its initiation. Detailed analysis of the WHI showed a significantly lower risk of coronary heart disease by estrogen therapy among women who were less than 10 years past the onset of menopause, and therefore, less time devoid of estrogen actions [12]. Reinforcing this analysis are the results of the ELITE trial specifically designed to test the hormone-timing hypothesis concerning cardiovascular risk in postmenopausal women [14]. Despite growing evidence validating the existence of a window of therapeutic opportunity for the cardiovascular benefits by estrogen, we still need to understand the mechanisms behind it. 

Vascular aging has been associated with a series of progressive functional alterations that can modify estrogen signaling in the arterial wall [25]. Considering that women enrolled in the clinical trials on hormone therapy and cardiovascular protections were middle-aged and had long been in menopause, it becomes difficult to separate the contribution of senescence from the long-term estrogen withdrawal to the adverse cardiovascular outcomes observed. In previous studies, we described that senescence dampens the beneficial effects of estrogen in aortas of OVX female mice [16]. However, we still do not know how senescence could modulate the risk-benefit ratio of estrogen use in arteries that are important for cerebral perfusion. Arteries of the distinct sizes and in distinct beds are regulated differently by intrinsic stimuli (including adrenergic stimulation) [26] and by the aging process [27]; therefore, it is of the utmost importance to understand the mechanisms of estrogens in the different senescent vascular beds, and more specifically in the cerebrovasculature. In this regard, we aimed to determine how the time to initiate estrogen treatment shapes estrogen-mediated effects in CCA of both non-senescent and senescent females. We used the OVX-SAMP8 as a model of menopause, based on our previous studies establishing OVX-SAMP8 as a suitable model to concomitantly study the effects of senescence and estrogen deficiency in middle-aged females [21]. 

In our preliminary studies in the carotid reactivity, we observed that basal response to estrogen might vary depending on mice strain (SAMR1 or SAMP8). When we compared the maximal contraction to Phe in carotid rings from intact (non-ovariectomized) SAMR1 and SAMP8 we observed that the maximal contractile response to Phe was markedly higher in intact SAMP8 (106 ± 5.2%) in comparison to intact SAMR1 (81 ± 1.8%, *p* < 0.0001), suggesting a detrimental effect of senescence in this vascular bed. Curiously, OVX did not modify the contractile response in SAMR1 (82.2 ± 2.3%, *p* = 0.812), while it deceased Phe contraction in SAMP8 (78.8 ± 1.8%, *p* < 0.001). Together, these results propose that estrogen plays a differential role in the control of vascular reactivity in young and senescent carotid arteries, even when at physiological levels.

A correlation between senescence and vascular dysfunction has been extensively described in men and women and much associated with decreased NO-mediated vasodilation [15,28]. Intact (non-ovariectomized) SAMP8 female mice presented a faster and time-dependent decrease in NO and increased oxidative stress compared to SAMR1 [29]. Estrogen withdrawal suppresses the involvement of NO in both groups [30], suggesting an overlap between aging and hormonal levels to control NO-mediated vascular function, similar to what is observed in postmenopausal women [31]. When females are non-senescent (SAMR1), estrogen therapy improves the vascular function of CCA (decreased Phe vasoconstriction) and augment NO bioavailability, via both upregulation of eNOS and decreased oxidative stress. Corroborating our data in SAMR1, several experimental studies in different vascular systems of young ovariectomized animal models show protective effects by estrogens in the aorta [5,32,33,34], mesenteric microvessels [8] and veins [9].

On the other hand, when females are senescent, estrogen treatment does not fully restore NO bioavailability in CCA. The reasons for this effect are unclear but may be related to the age-associated phenotypic changes in the vascular wall that diminish responsiveness [6,35] or modify signaling for estrogens [16]. In previous studies, we found that estrogens lose its ability to modulate eNOS expression, while significantly increases NADPH oxidase 1 (NOX1) expression and, consequently, the production of O_2_^−^ in aortas of OVX-SAMP8 mice [16].

In addition to NO, the cyclooxygenase (COX)-prostanoid cascade has also been implicated in the pathophysiology of vascular dysfunction during aging [36,37] and following estrogen withdrawal [7]. As revised by Hermenegildo and colleagues [38], estrogen increases the production of the COX-derived vasodilators PGI_2_ in different vascular beds—such as uterine, mesenteric, cerebral, and aortic—supporting additional benefit by this hormone in the female vasculature, whereas PGI_2_ metabolite levels decrease in postmenopausal women [39]. Moreover, PGI_2_ has been associated with the atheroprotective effect of estrogen [40]. In CCA of non-senescent SAMR1, COX-1 inhibition decreases Phe contraction, revealing an imbalance towards COX-1-derived vasoconstrictor prostanoids generation when estrogen is withdrawn by OVX. In contrast, COX-2 inhibition increases vasoconstriction in estrogen-treated SAMR1, suggesting an increased COX-2-derived vasodilator production by estrogen. Estrogen increased COX-2 mRNA expression in CCA of SAMR1 female, regardless of the timing for therapy initiation, which is associated with a marked increase of PGI_2_ concentration in the organ bath after Phe stimulation. Estrogen increases COX-2–mediated PGI_2_ production in both human endothelial cells [41] and mice artery [40]. 

On the other hand, the senescent carotids of SAMP8 show a different relationship between estrogens and prostanoids production. Our preliminary data using nonspecific COX inhibitor (indomethacin) agree with other studies describing an increase of COX-derived vasoconstrictor products by aging and estrogen withdrawal, as indomethacin decreases Phe-induced vasoconstriction in all SAMP8 groups. However, when we used selective COX-1 or COX-2 inhibitors, we did not see any change in Phe responses in CCA of OVX and E_2_E-treated SAMP8. Only in arteries of E_2_L females, both inhibitors decrease vasoconstrictor responses. These results suggest crosstalk between COX-1 and COX-2 in senescent arteries that maintain vasoconstriction when one or another is inhibited. Nevertheless, this compensation is missing in senescent females when they receive a late onset of estrogen therapy. The responses in E_2_L were associated with an increase of TXA_2_ synthase expression and TXA_2_ production after challenge with Phe. Augmented TXA_2_ production was previously reported in intact six months old SAMP8 female mice, and nonspecific COX inhibition corrected this parameter [29]. In postmenopausal women, estrogen increases platelet activation via TXA_2_ production [42]. In contrast, another study demonstrated that estrogen does not change TXA_2_ production in cultured human endothelial cells [43]. Together, these results suggest that the effects of estrogen on TXA_2_ production may depend on cell/tissue type and pathophysiological condition. 

The primary receptor responsible for the vascular actions of estrogens is the ERα, despite many studies describing vascular actions of ERβ and GPER [17]. Studies with ERα knockout [44] and Cre-lox recombinant endothelium-specific ERα knockout flox/flox (CreLoxP) mice [45] have demonstrated that ERα is necessary for estrogen-induced reendothelialization and vascular protection. Moreover, ERα activation has been associated with increased eNOS transcription and NO production [46], antioxidant effects [47], and regulation of prostanoids production [48], reinforcing that ERα activation is essential for most of the beneficial actions of estrogens in the vasculature. Expression of ERα is subject to complex regulation since the gene encoding this receptor subtype has a multiple promoter system, which produces distinct splicing variants during transcription. The two most-studied ERα isoforms are the 46Kd (ERα46) and the 36Kd (ERα36) receptors. ERα46 lacks the transcriptional activation function-1 (AF-1) in the N-terminus, while ERα36 misses both AF-1 and part of AF-2 in the C-terminal portion of the receptor. Thus, these variants may have limited transcriptional activity and can oppose genomic actions of the full-length 66Kd (ERα66) [17]. Disruption in estrogen signaling by upregulation of these splicing has been associated with cancer [20]. The pattern of ERα66 expression and its splicing might also be a determining factor in the aging process. It is known that alternative splicing is a typical post-transcriptional process in eukaryotes that can be associated with premature aging and associated diseases [49]. However, the contribution of alternative variants of ERα to women’s vascular health and disease remains an open question.

In the present study, we described for the first time that the CCA of female mice expresses both ERα46 and ERα36 variants. The expression of ERα36 mRNA was markedly lower relative to wild-type ERα66 in all experimental groups of SAMR1 mice. However, in senescent CCA late onset of estrogen treatment increased ERα36 expression to levels that were higher than the wild type receptors. The upregulation of ERα36 in E_2_L-SAMP8 was parallel by both increased TXA_2_ production and Phe vasoconstriction. Although we cannot provide direct evidence on the role of ERα36 on TXA_2_ production, due to the lack of specific agonist/antagonist for ER36, the fact that the expression of this variant is increased only in the group where we observed increased TXA_2_ release, points to a plausible relationship between these two pathways. Anti-ERα36 antibody treatment was shown to reduce estrogen-induced vasodilation in middle cerebral arteries from male rats [50]. Even though this study supports the vascular role of ERα36, the molecular mechanisms regulated by this specific ERα variant in the vascular wall warrant further examination. 

In summary, this study demonstrates that timing to initiate the treatment is a determining factor for the detrimental or beneficial effects of estrogens. More specifically, when arteries are senescent, late-onset estrogen therapy may represent a risk to the vasculature for increasing the production of contractile factors and vasoconstriction. Although we do not have direct evidence, the detrimental effects of estrogen are in parallel with an increase in expression of ERα36, which could plausibly modify estrogen signaling pathways towards vascular impairment. On average, women are living 20 years in menopause, and the use of estrogen in this population has been widely used to lessen problems associated with this natural process. This study draws our attention to the need for increasing our understanding of the mechanisms of action of estrogen receptors and their variants during cardiovascular aging in women. Because of the great controversy and the gap in the knowledge of the risks/benefits of estrogen use in the cardiovascular system, our results are of significant scientific impact. Besides, our results provide new insights into the regulation of estrogen receptors in aging and disease, which may eventually lead to the development of new regulatory molecules acting on estrogen receptors to improve vascular health in women and men.

## 5. Translational Perspective

The risk or benefit for estrogen use in menopausal women remain controversial, and the modulation of estrogen signaling in healthy, aged, and diseased vasculature is still unknown. In precision medicine times, we still do not know which population of women would benefit from the use of estrogen and in which one hormone therapy would pose a cardiovascular risk. This study shows that timing to initiate estrogen treatment is a determining factor for the detrimental or beneficial effects of estrogens and provide new insights into the regulation of estrogen receptors in aging and disease, which may eventually lead to the development of new regulatory molecules acting on estrogen receptors to improve vascular health in postmenopausal women.

## Figures and Tables

**Table 1:** Basic parameters (uterus weight and estrogen plasma concentration).

	SAMR1			SAMP8		
	OVX	E_2_E	E_2_L	OVX	E_2_E	E_2_L
**Uterus wet weight (mg/cm)**	30.1 ± 4.5	49.5 ± 5.4 *	56.1 ± 7.1 *	14.0 ± 1.7	42.4 ± 5.7 *	40.3 ± 5.5 *
**Uterus dry weight (mg/cm)**	7.9 ± 1.1	12.3 ± 1.0 *	13.9 ± 1.6 *	4.3 ± 0.8	12.1 ± 1.8 *	11.0 ± 1.2 *
**17β-estradiol (pg/mL)**	2.1 ± 0.5	4.2 ± 0.9 *	7.0 ± 1.2 *	1.2 ± 0.3	9.8 ± 1.4 *	7.4 ± 0.9 *

Values are means ± SEM of samples from 10 animals per group. The uterus weight was normalized by tibia length (mg tissue/cm tibia). The analysis was obtained in untreated ovariectomized mice (OVX), and ovariectomized mice under early-onset (E_2_E) or late-onset of 17β-estradiol treatment (E_2_L). * *p* < 0.05 vs. OVX.

## Supplementary Materials

The following are available online at https://www.mdpi.com/2073-4409/8/10/1217/s1, Figure S1: U46619-induced contraction; Figure S2: Acetylcholine (ACh) and Sodium Nitroprusside (SNP)-induced relaxation; Figure S3: Effects of reactive oxygen species (ROS) on Phenylephrine (Phe)-induced contractions; Figure S4: Effects of Cyclooxygenase (COX)-derived vasoactive metabolites on Phenylephrine (Phe)-induced contractions; Figure S5: Prostacyclin (PGI2) production; Figure S6: Thromboxane A2 (TXA2) production; Figure S7: Expression of the classical estrogen receptor (ERα66) in common carotid artery; Major Resources Table S1: Animal Model; Major Resources Table S2: Vascular Reactivity; Major Resources Table S3: Primer sequences for quantitative RT-PCR; Major Resources Table S4: Primer sequences for quantitative DNA methylation; Major Resources Table S5: Antibodies.

## Abbreviations

CCA	Common carotid artery
OVX	Ovariectomized
SAMP8	Senescence-accelerated mice prone–8
SAMR1	Senescence-accelerated mice resistant-1
E2	Estrogen
E2E	Early estrogen treatment
E2L	Late estrogen treatment
Phe	Phenylephrine
ACh	Acetylcholine
NPS	Sodium nitroprusside
ERα	Estrogen receptor alpha
COX-1	Cyclooxygenase 1
COX-2	Cyclooxygenase 2
PGI2	Prostacyclin
TXA2	Thromboxane A2
