# Supplementary material for: Late Onset of Estrogen Therapy Impairs Carotid Function of Senescent Females in Association with Altered Prostanoid Balance and Upregulation of the Variant ERα36
Source: Cells. 2019 Oct 8;8(10):1217. doi: 10.3390/cells8101217 (PMC6829869; doi:10.3390/cells8101217)
Supplement: Supplementary file 1 [file cells-08-01217-s001.pdf]

SUPPLEMENTARY DATA

Late onset of estrogen therapy impairs carotid function of senescent females in association with altered prostanoid balance and up-regulation of the variant ERα36

Tiago Januário Costa[a,b,c,d], Francesc Jiménez-Altayó[b], Cinthya Echem[a]; Eliana Hiromi Akamine[a], Rita Tostes[d], Elisabet Vila[b], Ana Paula Dantas[c]; Maria Helena Catelli de Carvalho[a].

Running title: Estrogen receptor signaling in senescent carotid

[a] Department of Pharmacology, Institute of Biomedical Sciences, University of Sao Paulo, Brazil.

[b] Facultat de Medicina, Departament de Farmacologia, Terapèutica i Toxicologia, Institut de Neurociències, Universitat Autònoma de Barcelona, Bellaterra, Spain.

[c] Group of Atherosclerosis and Coronary Disease, Institut Clinic del Torax, Institut d'Investigaciones Biomédiques August Pi I Sunyer (IDIBAPS), Barcelona, Spain.

[d] Pharmacology Department, Ribeirao Preto Medical School, University of Sao Paulo, Brazil.

Corresponding author:

Ana Paula Dantas, PhD.

Group of Atherosclerosis and Coronary Disease

Institut d'Investigaciones Biomédiques August Pi I Sunyer (IDIBAPS), Institut Clinic Cardiovascular.

Calle Casanova, 143, Cellex-2A.

08036. Barcelona, Spain.

e-mail: adantas@clinic.cat

MAJOR RESOURCES TABLES

Table S1. Animals.

Specie	Vendor or Source	Background Strain	Sex	Age
SAMR1	Inbred in-house	AKR/J	Female	6 months
SAMP8	Inbred in-house	AKR/J	Female	6 months

Table S2. Vascular Reactivity Studies.

Chemical	Catalog #	Company
9,11-Dideoxy-11α,9α-epoxymethanoprostaglandin $F_{2\alpha}$ (U46619)	D8174	Sigma-Aldrich
Acetylcholine chloride	A6625	Sigma-Aldrich
Sodium nitroprusside dihydrate	71778	Sigma-Aldrich
(*R*)-(-)-Phenylephrine hydrochloride	P-6126	Sigma-Aldrich
N_{ω}-Nitro-L-arginine methyl ester hydrochloride (L-NAME)	N5751	Sigma-Aldrich
COX1 inhibitor (SC560)	S2064	Sigma-Aldrich
COX2 inhibitor (NS398)	ab120295	Abcam
4-Hydroxy-TEMPO (tempol)	176141	Sigma-Aldrich

Table S3. Primer sequences for quantitative RT-PCR.

Gene (Pubmed ID)	Sequence (5′- 3′)
eNOS (NM_008713.4)	Forward: TGTCACTATGGCAACCAGCGT<br>Reverse: GCGCAATGTGAGTCCGAAAA
COX1 (NM_008969.3)	Forward: GAGCCGTGAGATGGGTGGGAGGG<br>Reverse: TGGATGTGCAATGCCAACGGCT
COX2 (NM_011198.3)	Forward: GTCAGGACTCTGCTCACGAAGGAAC<br>Reverse: ACAGCTCGGAAGAGCATCGCAG
PGI_2S (NM_008968.3)	Forward: CGGCTACCTGACCCTATATGGA<br>Reverse: GCCTGGCCCACAATTTCAAT
TXA_2S (NM_011539.3)	Forward: AAAGGAACCACCCCAAAGGT<br>Reverse: ACACGATCTTGGGCCTGACT
ERα (NM_007956.4)	Forward: TGCCTGGCTGGAGATTCTG Reverse: CTTCCCCGGGTGTTCCAT
CTERP (AB560752.1)	Forward: TGCCTGGCTGGAGATTCTG<br>Reverse: CTTCCCCGGGTGTTCCAT

Table 4. Primer sequences for quantitative DNA methylation.

Gene (Pubmed ID)	Sequence (5′-3′ Flanking Region)
ERS1-1 (AJ276597.1)	Forward: GGTTCACAGCCATCTCAGTTTC<br>Reverse: TTTGTATGTGGAGTGGCAGGG
ERS1-2 (AJ276597.1)	Forward: GAGAAGGAAGCTGTGCTGTTTTT<br>Reverse: AAAGCAAGCACTTCAGATGAGAC
ERS1-3 (AJ276597.1)	Forward: GAGCTGAAAGGTAGGAGAGCC<br>Reverse: CCAGACGAGTGGAGATGGAC
ERS1 -4 (AJ276597.1)	Forward: CAGGTTTGTCTAAGAGCAGAGGA<br>Reverse: CTTTGGTGTGAAGGGTCATGG

Table 5. Antibodies.

Target Antigen	Source (Company)	Catalog #	Working Concentration
Estrogen Receptor-alpha 36 isoform (ERA-36)	Alpha Diagnostic	ERA361-A	1:1000
ERα (MC-20)	Santa Cruz Biotechnology	SC-542	1:1000
Actin (Smooth Muscle)	Dako	M0851	1:2000

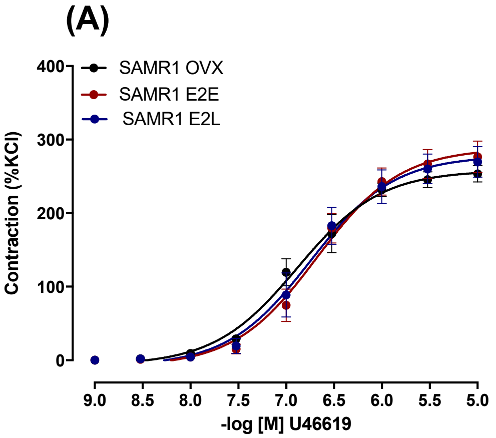

Figure S1. U46619-induced contraction. Concentration-response curves in endothelium-intact common carotid artery from SAMR1 (**A**) and SAMP8 (**B**) ovariectomized mice. Curves were obtained in vessels from untreated ovariectomized female mice (OVX), or ovariectomized under early-onset (E_2E) or late-onset of 17β-estradiol treatment (E_2L) treatment. Each point represents the mean ± SEM from 5-6 independent experiments. Differences in the fit of concentration response curves were determined by the extra sum-of-squares F.

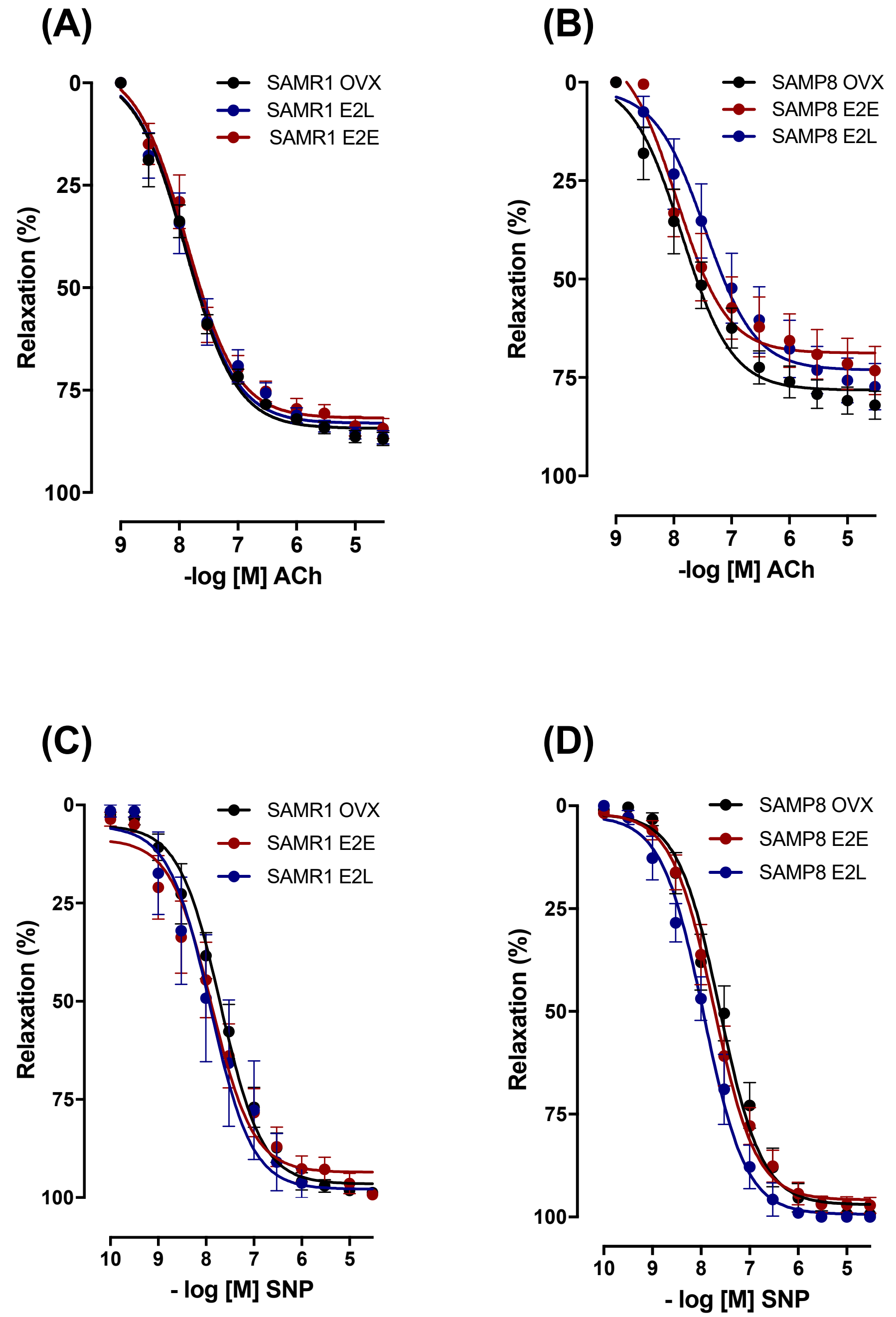

Figure S2 Acetylcholine (ACh) and Sodium Nitroprusside (SNP)-induced relaxation. Concentration-effect curves determined in endothelium-intact common carotids from SAMR1 (**A**,**C**) and SAMP8 (**B**,**D**) female mice. Curves were obtained in vessels from untreated ovariectomized female mice (OVX), or ovariectomized under early-onset (E_2E) or late-onset of 17β-estradiol treatment (E_2L) treatment. Each point represents the mean ± SEM from 6 independent experiments. Differences in the fit of concentration response curves were determined by the extra sum-of-squares F.

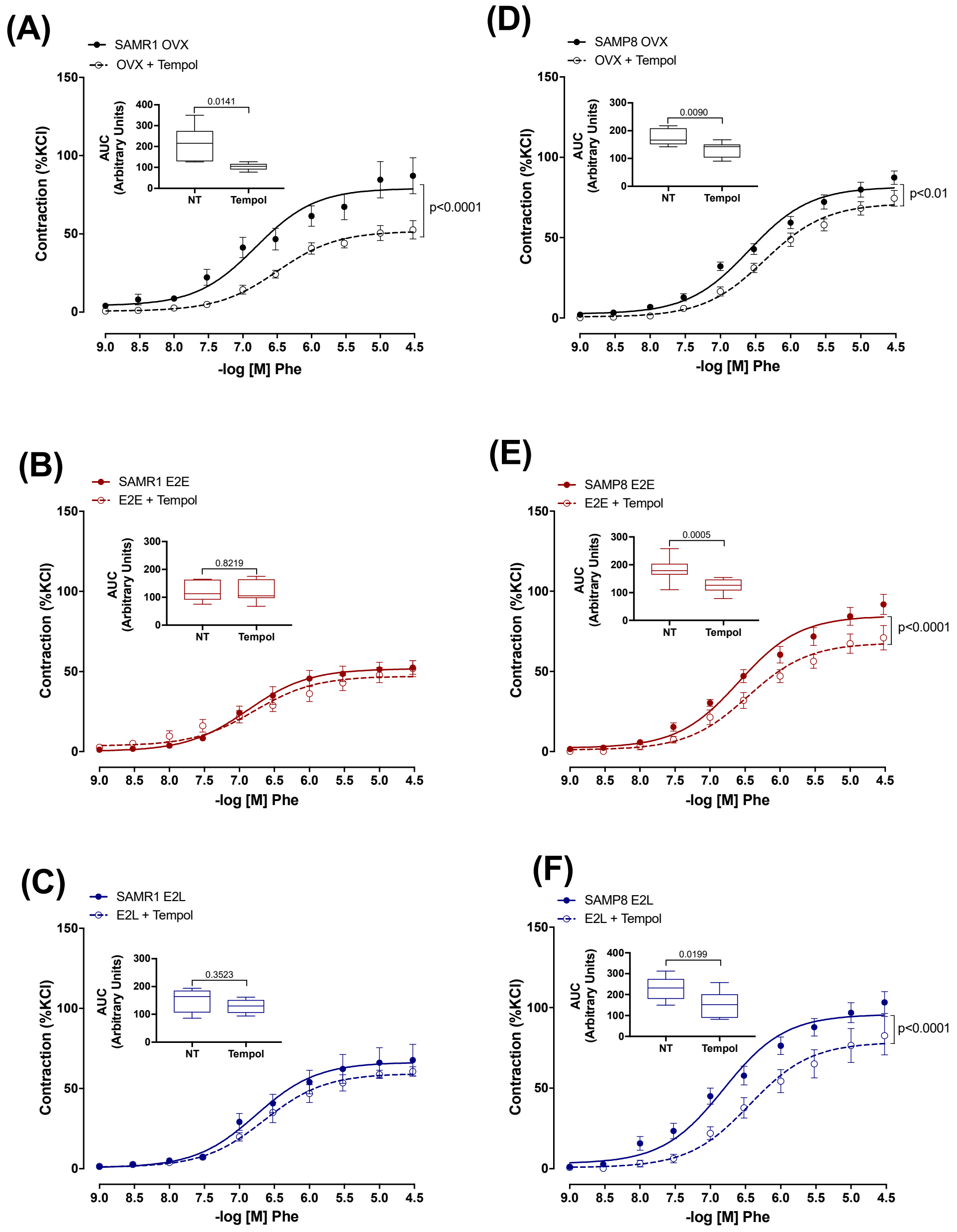

Figure 3. Effects of reactive oxygen species (ROS) on Phenylephrine (Phe)-induced contractions. Concentration-effect curves determined in endothelium-intact common carotids from female SAMR1 (A–C) and SAMP8 (D–F). The role of ROS in Phe-induced contraction was assessed with Tempol (10^{-5} M). Inset graphs represent the area under the curve (AUC) of Phe curves of untreated carotids (NT) or arteries receiving Tempol. Curves were obtained in vessels from untreated ovariectomized mice (OVX – A and D), ovariectomized mice under early-onset (E_2E – B and E) or late-onset (E_2L – C and F) 17β-estradiol treatment. Each point represents the mean ± SEM from 8–9 independent experiments. Differences in the fit of concentration response curves were determined by the extra sum-of-squares F. The dependence of data on the onset of estrogen therapy (none, early or late) in SAMR1 or SAMP8 was analyzed by one-way ANOVA with Bonferroni's post-test. P values and comparisons are expressed next to the curves and on top of AUC bar graphs. Significance is considered when $p < 0.05$.

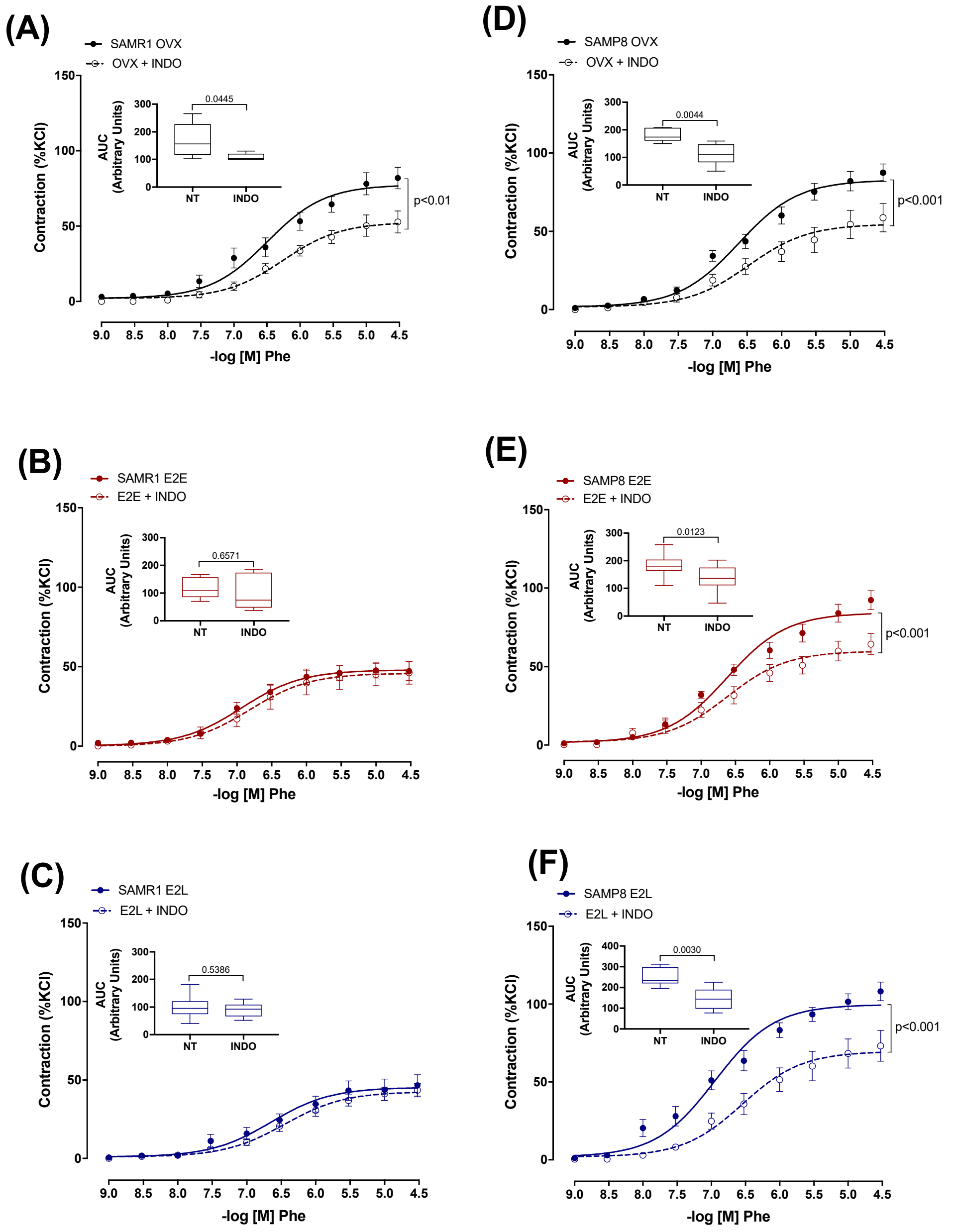

Figure 6. Effects of Cyclooxygenase (COX)-derived vasoactive metabolites on Phenylephrine (Phe)-induced contractions. Concentration-effect curves determined in endothelium-intact common carotids from female SAMR1 (**A**–**C**) and SAMP8 (**D**–**F**). The role of COX metabolites in Phe-induced contraction was assessed with the unspecific COX inhibitor, Indomethacin (INDO, 10^{-5} M). Inset graphs represent the area under the curve (AUC) of Phe curves of untreated carotids (NT) or arteries receiving INDO. Curves were obtained in vessels from untreated ovariectomized mice (OVX – A and D), ovariectomized mice under early-onset (E_2E – B and E) or late-onset (E_2L – C and F) 17β-estradiol treatment. Each point represents the mean ± SEM from 8–9 independent experiments. Differences in the fit of concentration response curves were determined by the extra sum-of-squares F. The dependence of data on the onset of estrogen therapy (none, early or late) in SAMR1 or SAMP8 was analyzed by one-way ANOVA with Bonferroni's post-test. P values and comparisons are expressed next to the curves and on top of AUC bar graphs. Significance is considered when $p<0.05$.

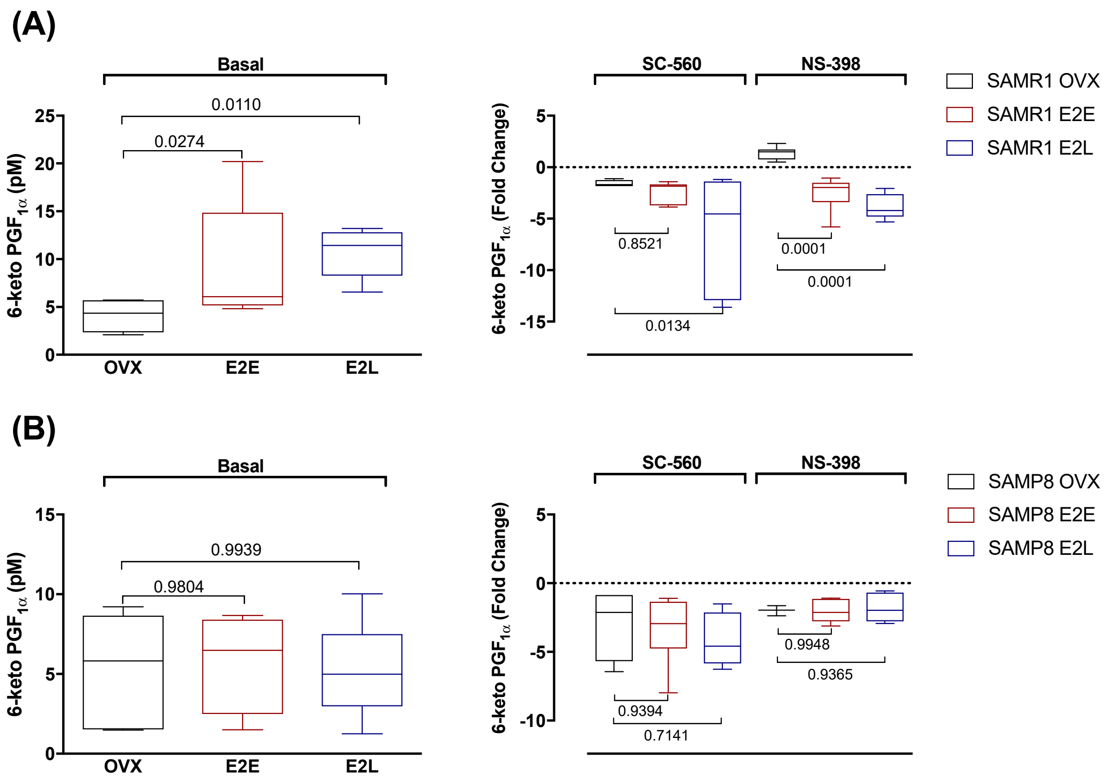

Figure S5. Prostacyclin (PGI2) production. Levels of PGI2 metabolite, 6-KetoPGF2α, in Krebs solution after concentration-effect curves to phenylephrine in the presence of vehicle (Basal), COX-1 inhibitor (SC-560, 10^{-5} M) or COX-2 inhibitor (NS-398, 10^{-6} M) in endothelium-intact common carotids from female SAMR1 **(A)** and SAMP8 **(B)** female mice. Basal levels of 6-KetoPGF2α are expressed as a concentration in pM (left panels), and changes in of 6-KetoPGF2α concentration after specific COX inhibition are expressed as Log2 fold change related to basal (right panels). Experimental Groups: untreated ovariectomized (OVX), early 17β-estradiol treatment (E_2E) and late 17β-estradiol treatment (E_2L). Results represent the mean ± SEM from 5–8 independent experiments. The dependence of data on the onset of estrogen therapy (none, early or late) in SAMR1 or SAMP8 was analyzed by one-way ANOVA with Bonferroni's post-test. P values and comparisons are expressed on top of bar graphs. Significance is considered when $p < 0.05$.

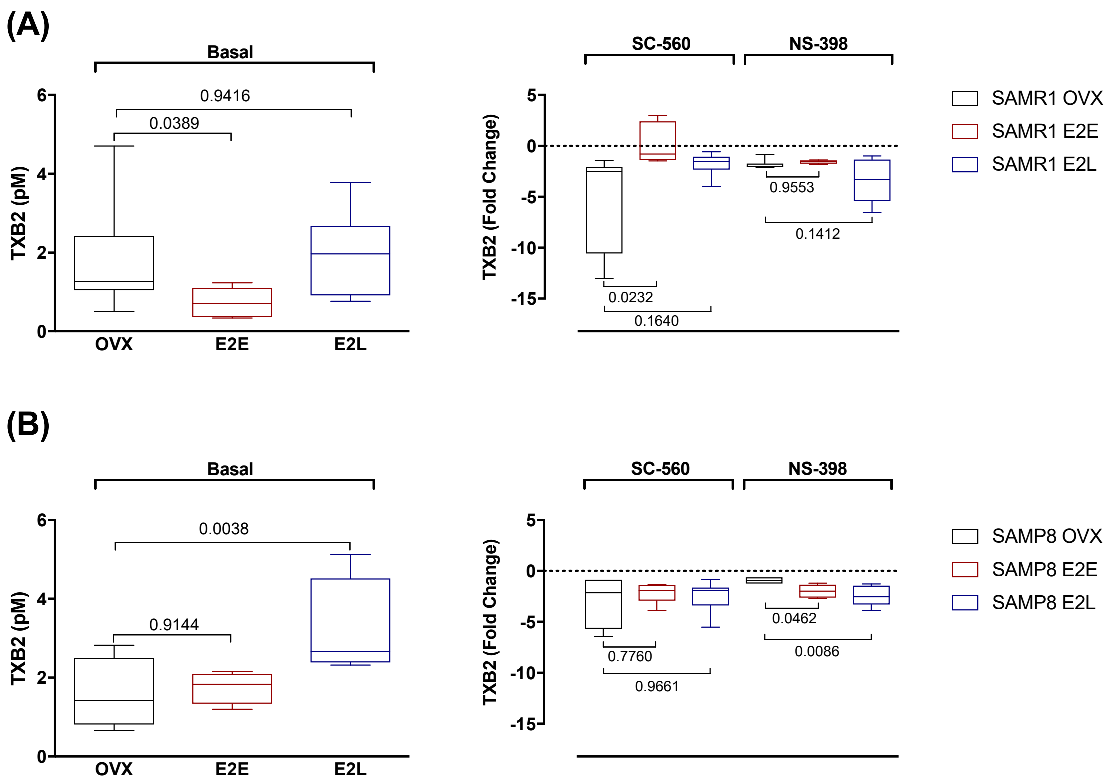

Figure S6. Thromboxane A2 (TXA2) production. Levels of TXA2 metabolite, TXB2, in Krebs solution after concentration-effect curves to phenylephrine in the presence of vehicle (Basal), COX-1 inhibitor (SC-560, 10^{-5}M) or COX-2 inhibitor (NS-398, 10^{-6}M) in endothelium-intact common carotids from female SAMR1 **(A)** and SAMP8 **(B)** female mice. Basal levels of TXB2 are expressed as a concentration in pM (left panels), and changes in of TXB2 concentration after specific COX inhibition are expressed as Log2 fold change related to basal (right panels). Experimental Groups: untreated ovariectomized (OVX), early 17β-estradiol treatment (E_2E) and late 17β-estradiol treatment (E_2L). Differences in the fit of concentration response curves were determined by the extra sum-of-squares F. The dependence of data on the onset of estrogen therapy (none, early or late) in SAMR1 or SAMP8 was analyzed by one-way ANOVA with Bonferroni's post-test. P values and comparisons are expressed on top of bar graphs. Significance is considered when $p < 0.05$.

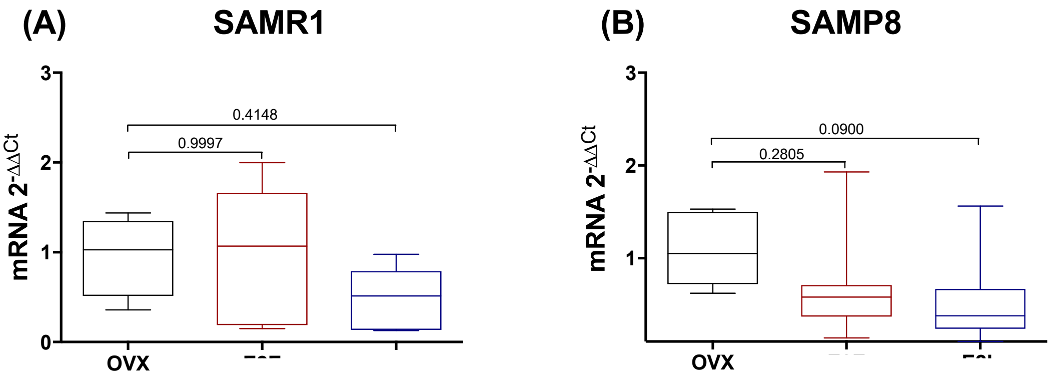

Figure VII. Expression of the classical estrogen receptor (ERα66) in common carotid artery. mRNA expression was determined in arteries of ovariectomized (OVX) SAMR1 (A) and SAMP8 (B) mice, OVX mice under early-onset 17β-estradiol treatment (E_2E), and OVX mice under late-onset 17β-estradiol treatment (E_2L). Results were normalized to β-actin expression and are expressed relative to the OVX groups. The dependence of data on the onset of estrogen therapy (none, early or late) in SAMR1 or SAMP8 was analyzed by one-way ANOVA with Bonferroni's post-test. Bar graphs represent the mean ± SEM from 5–6 independent experiments. P values and comparisons are expressed on top of bar graphs. Significance is considered when $p < 0.05$.
